# Expanded clinical phenotype and untargeted metabolomics analysis in *RARS2*-related mitochondrial disorder: a case report

**DOI:** 10.1186/s12883-024-03571-w

**Published:** 2024-03-04

**Authors:** Ameya S. Walimbe, Keren Machol, Stephen F. Kralik, Elizabeth A. Mizerik, Yoel Gofin, Mir Reza Bekheirnia, Charul Gijavanekar, Sarah H. Elsea, Lisa T. Emrick, Fernando Scaglia

**Affiliations:** 1 Division of Pediatric Neurology and Developmental Neuroscience, Department of Pediatrics, Baylor College of Medicine, Houston, TX 77030 USA; 2 Texas Children’s Hospital, Houston, TX 77030 USA; 3 Department of Molecular and Human Genetics, Baylor College of Medicine, Houston, TX 77030 USA; 4 Department of Radiology, Baylor College of Medicine, Houston, TX 77030 USA; 5 School of Medicine, Faculty of Medical and Health Sciences, Tel Aviv University, Tel Aviv, Israel; 6 Genetics Institute, Meir Medical Center, Kfar Saba, Israel; 7 BCM-CUHK Center of Medical Genetics, Prince of Wales Hospital, Hong Kong SAR, China

**Keywords:** *RARS2*, Mitochondrial disease, Dysmorphic features, Lennox-Gastaut Syndrome, Untargeted metabolomics analysis

## Abstract

**Background:**

*RARS2*-related mitochondrial disorder is an autosomal recessive mitochondrial encephalopathy caused by biallelic pathogenic variants in the gene encoding the *mitochondrial arginyl-transfer RNA synthetase 2* (*RARS2*, MIM *611524, NM_020320.5). RARS2 catalyzes the transfer of L-arginine to its cognate tRNA during the translation of mitochondrially-encoded proteins. The classical presentation of *RARS2*-related mitochondrial disorder includes pontocerebellar hypoplasia (PCH), progressive microcephaly, profound developmental delay, feeding difficulties, and hypotonia. Most patients also develop severe epilepsy by three months of age, which consists of focal or generalized seizures that frequently become pharmacoresistant and lead to developmental and epileptic encephalopathy (DEE).

**Case presentation:**

Here, we describe a six-year-old boy with developmental delay, hypotonia, and failure to thrive who developed an early-onset DEE consistent with Lennox-Gastaut Syndrome (LGS), which has not previously been observed in this disorder. He had dysmorphic features including bilateral macrotia, overriding second toes, a depressed nasal bridge, retrognathia, and downslanting palpebral fissures, and he did not demonstrate progressive microcephaly. Whole genome sequencing identified two variants in *RARS2*, c.36 + 1G > T, a previously unpublished variant that is predicted to affect splicing and is, therefore, likely pathogenic and c.419 T > G (p.Phe140Cys), a known pathogenic variant. He exhibited significant, progressive generalized brain atrophy and *ex vacuo* dilation of the supratentorial ventricular system on brain MRI and did not demonstrate PCH. Treatment with a ketogenic diet (KD) reduced seizure frequency and enabled him to make developmental progress. Plasma untargeted metabolomics analysis showed increased levels of lysophospholipid and sphingomyelin-related metabolites.

**Conclusions:**

Our work expands the clinical spectrum of *RARS2*-related mitochondrial disorder, demonstrating that patients can present with dysmorphic features and an absence of progressive microcephaly, which can help guide the diagnosis of this condition. Our case highlights the importance of appropriate seizure phenotyping in this condition and indicates that patients can develop LGS, for which a KD may be a viable therapeutic option. Our work further suggests that analytes of phospholipid metabolism may serve as biomarkers of mitochondrial dysfunction.

**Supplementary Information:**

The online version contains supplementary material available at 10.1186/s12883-024-03571-w.

## Introduction

*RARS2* (MIM*611524, NM_020320.5) is a nuclear gene that encodes the mitochondrial arginyl-transfer RNA synthetase 2, an aminoacyl-tRNA synthetase (aaRS), which charges human mitochondrial tRNA with arginine during the translation of mitochondrial proteins required for ATP production. Biallelic pathogenic variants in *RARS2* cause an autosomal recessive mitochondrial encephalopathy known as *RARS2*-related mitochondrial disorder [1]. Approximately seventy patients with this condition have been reported in the literature [2–6]. The classic presentation of *RARS2*-related mitochondrial disorder includes pontocerebellar hypoplasia (PCH), epilepsy, profound developmental delay, feeding difficulties, progressive microcephaly, and hypotonia. Various metabolic derangements have also been observed, including metabolic acidosis, hypoglycemia, hyperammonemia, abnormalities in acylcarnitine profile and urine organic acid analysis, and lactic acidosis in blood, cerebrospinal fluid (CSF), and urine [3–5].

Most patients with *RARS2*-related mitochondrial disorder also develop severe epilepsy, experiencing their first seizure by three months of age [6]. A wide variety of focal, multifocal, or generalized seizure types can be observed in *RARS2*-related mitochondrial disorder [6]. The most common of these are myoclonic, focal clonic, or generalized tonic–clonic seizures. However, patients may experience several other seizure types, including focal with impaired awareness, atonic, tonic, or absence seizures, and they may develop episodes of convulsive or non-convulsive status epilepticus [6]. Epilepsy in *RARS2*-related mitochondrial disorder often becomes pharmacoresistant and evolves to developmental and epileptic encephalopathy (DEE) [1–6].

We describe a six-year-old boy with *RARS2*-related mitochondrial disorder who developed an early-onset DEE consistent with Lennox-Gastaut syndrome (LGS), which has not been reported before. He presented with progressive, generalized brain atrophy and lacked PCH on brain MRI. Furthermore, he exhibited several dysmorphic features on exam that have not been previously observed. A ketogenic diet (KD) reduced seizure frequency and allowed him to progress developmentally. Untargeted metabolomics analysis demonstrated elevations in compounds involved in lysophospholipid and sphingomyelin metabolism. Our report expands the clinical spectrum of *RARS2*-related disorder and suggests that patients may develop LGS, for which a KD may be beneficial. In addition, our patient’s metabolomics analysis may shed light on potential biomarkers associated with mitochondrial disorders.

## Case presentation

**Fig. 1:**
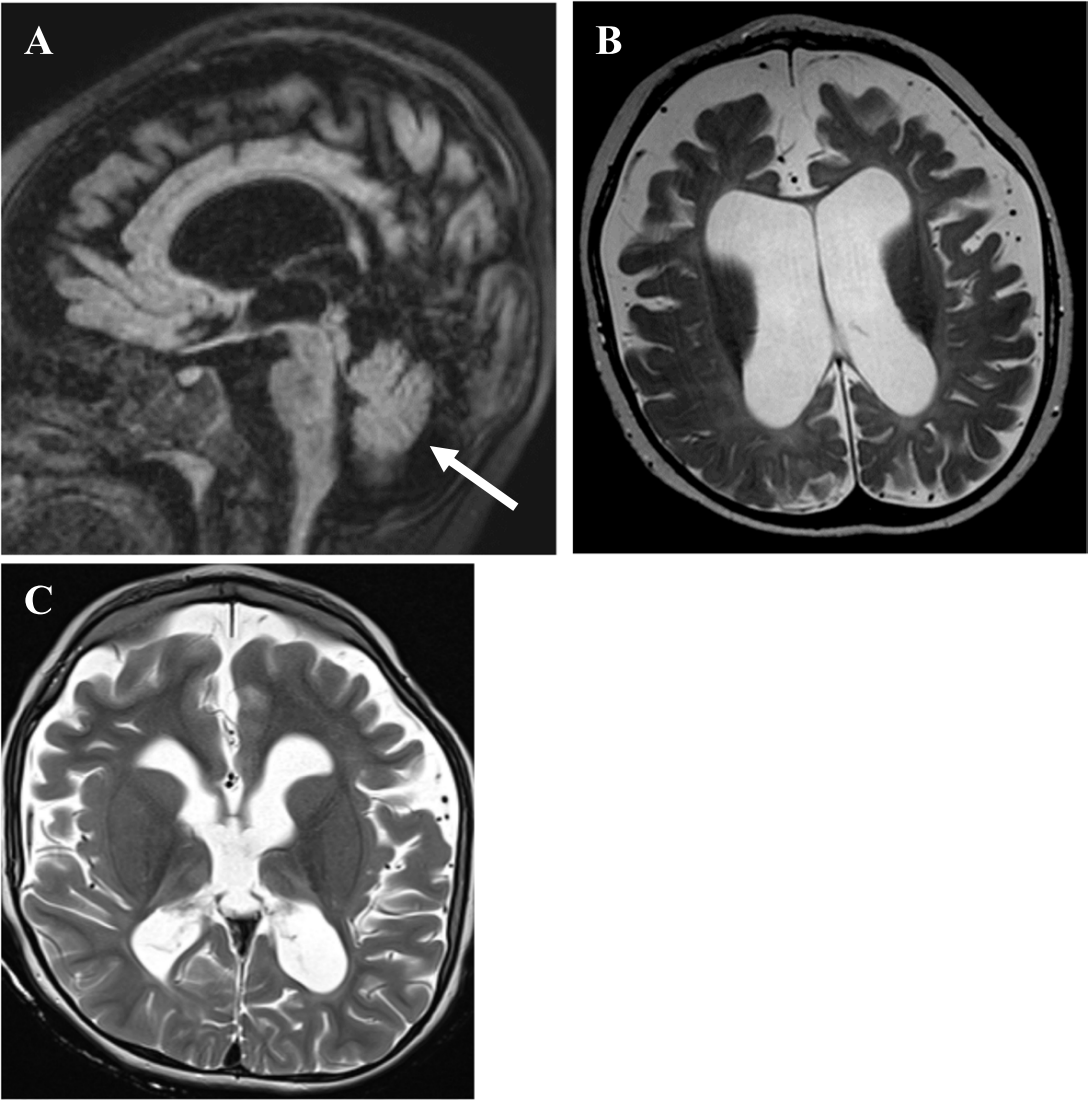
Brain MRIs of a patient with *RARS2*-related Mitochondrial Disorder. (Top Left) Sagittal FLAIR image of brain MRI at twelve months of age showing large subarachnoid spaces, ventriculomegaly, and generalized brain atrophy. There is a minimal reduction in brainstem size, and the volume of the cerebellum is preserved (white arrow). (Top Right) Axial T2W image at twelve months of age demonstrating *ex vacuo* enlargement of the ventricles and widening of the subarachnoid spaces. (Bottom Left) Axial T2W image at four years and six months of age demonstrating new T2 hyperintense signals in the bilateral thalami in addition to generalized volume loss, thinning of the corpus callosum, *ex vacuo* enlargement of the ventricles, and widening of the subarachnoid spaces

The patient was born uneventfully at thirty-nine weeks gestation. At one month of age, he developed myoclonia of his right eyelid. The myoclonia was found to represent focal motor epilepsy and improved with phenobarbital. At four months of age, he developed infantile spasms and experienced a developmental plateau. A brain MRI at seven months of age was notable for large subarachnoid spaces, ventriculomegaly, and brain atrophy. Family history was negative for epilepsy or intellectual disability. However, by nine months of age, he showed minimal alertness and could not babble, orient to sounds, reach for toys, pull to stand, or interact with others. At twelve months of age, he had limited social interactions, demonstrated hypotonia and poor truncal stability, lacked head control, and was unable to stand but could move his extremities against gravity. His head circumference (HC), measured as the occipitofrontal circumference (OFC), was at the 87th percentile (WHO Boys HC 0–5 years) at twelve months of age. Brain MRI was notable for large subarachnoid spaces, ventriculomegaly, generalized brain atrophy, and a minimal reduction in brainstem size, but it demonstrated a normal cerebellar volume (Fig. 1 Top Left, Top Right).

A 24-h EEG demonstrated a modified hypsarrhythmic pattern with multifocal spikes greatest posteriorly, which, together with his clinical course, indicated West Syndrome (Supplementary Table 1). Spasms persisted despite the use of ACTH, prednisolone, and vigabatrin; thus, treatment with zonisamide and clobazam was initiated. Screening for a KD was performed and demonstrated a normal serum lactate at 0.7 mmol/L (reference: 0.2–1.7 mmol/L) but revealed carnitine deficiency with a free carnitine of 15 µmol/L (reference: 25–54 µmol/L). KD was deferred in favor of optimizing anti-seizure medications (ASMs). However, carnitine supplementation was initiated with a dose of 100 mg/kg/day. He experienced improvements in tone and began to kick his legs and move his arms, enabling him to participate in physical and occupational therapies. He developed significant oral phase dysphagia related to his underlying mitochondrial disorder that led to poor weight gain. Subsequently, at twenty-six months and one week of age, he was diagnosed with failure to thrive (FTT) with a weight of 10.7 kg (3rd percentile, CDC Boys 2–20). His height was 89.6 cm (64th percentile, CDC Boys 2–20), and his HC was 47 cm (22nd percentile, WHO Boys HC 0–5 years).

By three years and six months of age, his spasms occurred in clusters approximately twice per day. The addition of cannabidiol to his existing ASM regimen reduced the frequency of his spasms to sporadic events. However, he developed new episodes of shaking and teeth clenching, which were found to be caused by multifocal myoclonic seizures. Myoclonic seizures occurred approximately three times a month but were concerning for evolution of his West Syndrome to LGS. As such, rufinamide was added to his ASM regimen. He continued to show some developmental gains. He could recognize when others were talking to him, turn his head, and show interest in others. He continued to have difficulty gaining weight (12.7 kg at four years of age, < 5th percentile, z-score -2.17). Thus, a gastrostomy tube was placed with subsequent improvement in weight gain.

He was admitted to the intensive care unit (ICU) at four years and six months of age in refractory status epilepticus triggered by a rhinoviral infection. Evaluation by the Genetics consult service demonstrated downslanting palpebral fissures, bilateral macrotia, a depressed nasal bridge, retrognathia, and bilateral overriding second toes. A 24-h EEG showed numerous tonic and myoclonic seizures, frequent generalized spike-and-wave discharges at 1.5–2.5 Hz, and generalized paroxysmal fast activity while asleep, which together indicated LGS. A brain MRI was notable for progressive, generalized volume loss of the brain and signal abnormality in bilateral cerebral hemispheres (Fig. 1 Bottom Left). A next-generation sequencing panel for 302 epilepsy genes was obtained but was non-diagnostic. Given the patient’s critical condition and the high risk of clinical decompensation and death, rapid trio whole genome sequencing (WGS) was requested to analyze variants in coding and non-coding regions as well as pathogenic variants and single deletions in mitochondrial DNA (mtDNA). The test was performed by Baylor Genetics (BG). WGS did not detect any pathogenic copy number variants (CNVs) but returned positive for two single nucleotide variants (SNVs) in *RARS2*, c.419 T > G (p.Phe140Cys) and c.36 + 1G > T. The first variant, c.419 T > G (p.Phe140Cys), is a previously reported pathogenic missense variant inherited from the patient’s father, and c.36 + 1G > T is an unpublished variant inherited from his mother. The c.419 T > G (p.Phe140Cys) variant (ClinVar Variation ID: 215055, Accession SCV000992715.2) has been observed in gnomAD with a frequency of 0.010%. It has an inconclusive theoretical prediction score (CADD: 29.500) but affects a highly conserved residue. The c.36 + 1G > T variant is expected to disrupt a canonical splicing donor site in intron 1, which may lead to splicing defects such as exon skipping or intron retention and, thereby, disrupt normal protein function (SpliceAI: 0.970). It is, therefore, predicted to be deleterious (CADD: 33) and is reported as likely pathogenic (ClinVar Variation ID: 2442235, Accession SCV003835680.1). The level of growth differentiation factor 15 (GDF15) was elevated at 933 pg/mL (reference ≤ 750 pg/mL). Plasma untargeted metabolomics analysis was performed by BG on an ultrahigh-performance liquid chromatography-tandem mass spectrometry platform, as previously reported [7]. The test revealed elevations of carnitine metabolites due to carnitine supplementation and increased levels of analytes in the lysophospholipid and sphingomyelin subpathways (Table 1).

**Table 1:** Metabolomic perturbations in complex lipid pathways in a patient with *RARS2*-related mitochondrial disorder

BIOCHEMICAL	Z-score	CATEGORY	HMDB_ID
1-docosapentaenoyl-GPC (22:5n3)	3.6	Lysophospholipid	HMDB0010403
1-eicosapentaenoyl-GPC (20:5)	3.2	Lysophospholipid	HMDB0010397
1-stearoyl-GPI (18:0)	2.0	Lysophospholipid	HMDB0240261
1-dihomo-linolenoyl-GPC (20:3n3 or 6)	2.0	Lysophospholipid	HMDB0010394
1-docosahexaenoyl-GPC (22:6)	2.3	Lysophospholipid	HMDB0010404
1-stearoyl-2-oleoyl-GPC (18:0/18:1)	2.0	Phosphatidylcholine	HMDB0008038
1-oleoyl-2-dihomo-linolenoyl-GPC (18:1/20:3)	1.9	Phosphatidylcholine	HMDB0008113
1-stearoyl-2-docosapentaenoyl-GPC (18:0/22:5n3)	3.9	Phosphatidylcholine	HMDB0008056
1-palmitoyl-2-eicosapentaenoyl-GPC (16:0/20:5)	3.3	Phosphatidylcholine	HMDB0007984
1-stearoyl-2-dihomo-linolenoyl-GPC (18:0/20:3n3 or 6)	3.1	Phosphatidylcholine	HMDB0008047
phosphatidylcholine (18:0/20:5, 16:0/22:5n6)	2.8	Phosphatidylcholine	HMDB0008050, HMDB0007989
1-stearoyl-2-docosahexaenoyl-GPC (18:0/22:6)	2.2	Phosphatidylcholine	HMDB0008057
1-palmitoyl-2-docosahexaenoyl-GPC (16:0/22:6)	2.2	Phosphatidylcholine	HMDB0007991
1-stearoyl-2-docosahexaenoyl-GPE (18:0/22:6)	2.6	Phosphatidylethanolamine	HMDB0009012
1-palmitoyl-2-docosahexaenoyl-GPE (16:0/22:6)	2.2	Phosphatidylethanolamine	HMDB0008946
N-stearoyl-sphingosine (d18:1/18:0)	2.4	Ceramides	HMDB0004950
palmitoyl dihydrosphingomyelin (d18:0/16:0)	2.1	Dihydrosphingomyelins	HMDB0010168
sphingomyelin (d18:0/18:0, d19:0/17:0)	2.0	Dihydrosphingomyelins	HMDB0012087
stearoyl sphingomyelin (d18:1/18:0)	2.7	Sphingomyelins	HMDB0001348
palmitoyl sphingomyelin (d18:1/16:0)	2.4	Sphingomyelins	HMDB0010169
sphingomyelin (d18:1/24:1, d18:2/24:0)	2.2	Sphingomyelins	HMDB0012107
behenoyl sphingomyelin (d18:1/22:0)	2.2	Sphingomyelins	HMDB0012103
lignoceroyl sphingomyelin (d18:1/24:0)	2.1	Sphingomyelins	HMDB0011697

The genomic findings, in conjunction with his clinical picture and elevated levels of GDF15, were thought to confirm the diagnosis of *RARS2*-related mitochondrial disorder. Given the diagnosis of a mitochondrial disorder, ubiquinol supplementation was initiated with a dose of 8 mg/kg/day, and zonisamide was discontinued due to a lack of efficacy. Given his failure of several ASMs and the refractory nature of his epilepsy, a KD was implemented at four years and nine months of age. A KD reduced spasm frequency to rare events and markedly increased his alertness and awareness. He showed improvements in his gross motor skills and gained the ability to stand, which enabled him to better engage in therapies. HC at four years and nine months was 49.4 cm (20th percentile, WHO Boys HC 0–5). Repeat EEG, after initiation of a KD, showed only one generalized myoclonic-tonic seizure and one cluster of epileptic spasms. He has rare myoclonic-tonic seizures and continues to make developmental gains.

## Discussion and conclusions

RARS2 is an aminoacyl tRNA synthetase that charges cognate tRNAs with L-arginine during mitochondrial protein translation [1]. The pathomechanism of disease in *RARS2*-related mitochondrial disorder is caused by loss of function biallelic variants in *RARS2* that lead to reduced RARS2 expression and enzymatic activity [8, 9]. The classic findings of PCH, progressive microcephaly, developmental delay, and severe epilepsy occur because tissues with high metabolic demands, including the brain, lose the ability to effectively generate adenosine triphosphate (ATP) via oxidative phosphorylation [1, 10]. As such, patients with *RARS2*-related mitochondrial disorder frequently demonstrate structural abnormalities on brain MRI (Supplementary Table 1). Forty-eight percent of patients have been reported to have PCH [11], while 71% demonstrate supratentorial defects such as cerebral atrophy, thinning of the corpus callosum, or ventriculomegaly [3]. Cerebral atrophy leads to loss of cerebral white matter, and this finding is observed in several patients with *RARS2*-related mitochondrial disorder (Supplementary Table 1). The exact consequence of white matter loss in *RARS2*-related mitochondrial disorder is unclear. However, a selective loss of white matter tracts is observed in patients with aaRS disorders more generally [12]. Moreover, in other forms of leukoencephalopathy, the degree of white matter involvement correlates with phenotypic severity. The degree of white matter loss in *RARS2*-related mitochondrial disorder may, therefore, correspond with the level of encephalopathy or epilepsy [13, 14]. Cerebral atrophy may also reduce the amount of cortical gray matter, which can be a late finding in *RARS2*-related mitochondrial disorder (Supplementary Table 1). Gray matter abnormalities frequently cause seizures. Thus, cortical volume loss with or without cerebral atrophy may contribute to epilepsy in *RARS2*-related mitochondrial disorder. Our patient’s brain MRI at four years and six months of age was notable for significantly progressive, generalized volume loss of the brain. It also demonstrated *ex vacuo* dilatation of the supratentorial ventricular system, which is likely secondary to the global brain atrophy and a thinned corpus callosum, and it did not show PCH. Generalized atrophy and the absence of PCH have been described previously, so our patient’s MRI findings are consistent with those observed in other patients and likely contributed to his neurologic presentation.

Over fifty *RARS2* variants have been reported (Supplementary Table 1). Approximately 60% are missense variants and 20% are splice-site variants [3]. The remainder is a combination of small deletions or insertions, nonsense pathogenic variants, translation initiation codon variants, and frameshift pathogenic variants [2–4, 15]. Rapid WGS was performed in our patient given his critical status and high risk of deterioration and death and revealed two variants in *RARS2*, c.419 T > G, (p.Phe140Cys) and c.36 + 1G > T. c.419 T > G. The first variant (p.Phe140Cys) is a previously published pathogenic missense variant, and c.36 + 1G > T is an unpublished variant that is predicted to disrupt a splicing donor site in intron 1 and is, thereby, predicted to be deleterious. Both variants likely reduce RARS2 expression or activity, thereby leading to disease via a loss-of-function mechanism. Our report, therefore, expands the spectrum of known pathogenic variants in this condition.

Our patient experienced severe epilepsy, first developing focal motor seizures and subsequently West Syndrome, which ultimately evolved into LGS. The diagnosis of LGS was based on his tonic and myoclonic seizures and an EEG, which demonstrated a diffuse slow spike and wave pattern and generalized paroxysms of fast activity in sleep. Our report is the first to document LGS in *RARS2*-related mitochondrial disorder. However, given the prevalence and severity of epilepsy in this condition, other unreported patients with *RARS2*-related mitochondrial disorder may have LGS. Given the diagnosis of LGS and the pharmacoresistance of his epilepsy, a KD was implemented at four years and nine months of age, which dramatically reduced his seizure frequency, improved his alertness, and facilitated his acquisition of developmental milestones. The degree of seizure control experienced by our patient has not been seen in other patients with *RARS2*-related mitochondrial disorder treated with a KD (Supplementary Table 1). Valles-Ibanez et al. report a girl who developed a progressive movement disorder by nine months of age that subsequently progressed to a myoclonic DEE. Despite having tried over ten ASMs in addition to a KD, this patient suffers from daily atypical absence, myoclonic, and atonic seizures [6]. Ngoh et al. describe two male siblings with *RARS2*-related mitochondrial disorder who developed West syndrome and refractory epilepsy requiring several ASMs [16]. Treatment with a KD led to a modest reduction in seizure frequency in one sibling, while the other experiences daily seizures despite multiple ASMs and a KD. A female patient reported by Nishri et al. developed a severe DEE with focal, multifocal, and myoclonic seizures, which, too, was not well-controlled with multiple ASMs in addition to a KD [17]. More studies are needed to determine the exact efficacy of a KD in *RARS2*-related mitochondrial disorder. However, several clinical trials have demonstrated its benefit in managing epilepsy for other mitochondrial disorders [18–21]. In a group of fourteen patients with mitochondrial disorders treated with a KD, 10/14 patients demonstrated a greater than 50% reduction in seizure frequency, and 7/14 achieved complete seizure freedom [18]. Another study of twenty patients with LGS and mitochondrial complex I deficiency who received either a KD or Modified Atkins Diet (MAD) reported that seven patients experienced at least a 50% reduction in seizure frequency after two years of treatment [19]. A systematic review by Zweers et al. identified eight patients with defects in other genes associated with mitochondrial disease, including *POLG*, *SLC25A12*, *MT-TL1*, and *MTO1*, of whom seven experienced control of therapy-refractory epilepsy with a KD [20]. Ketosis achieved through such dietary therapies (DT) has been shown to reduce oxidative damage in the brain, decrease levels of mitochondrial reactive oxygen species, and increase the amount of glutathione peroxidase in hippocampal neurons [21]. Thereby, DT may exert neuroprotective and anticonvulsant effects by improving mitochondrial function, and their benefits may extend beyond epilepsy. Indeed, one patient with *lysyl tRNA synthetase 2 (KARS2)*–related mitochondrial disorder showed an improvement in psychomotor regression on a KD [22]. As our patient experienced fewer seizures with a KD and improved seizure control enabled him to make developmental gains, a KD may have a role in treating both epilepsy and developmental delay in *RARS2*-related mitochondrial disorder. The experience with our patient also emphasizes the need for careful seizure phenotyping in *RARS2*-related mitochondrial disorder, as a diagnosis of LGS may open new treatment options for patients, including a KD.

Metabolic abnormalities have been frequently reported in *RARS2*-related mitochondrial disorder, and the most common of these is lactic acidosis in serum and CSF [3]. Lactic acidosis has been observed in approximately 40% of patients with *RARS2*-related mitochondrial disorder and is likely a result of impaired mitochondrial respiration and oxidative ATP production [3, 12, 23]. Interestingly, we did not detect lactic acidosis in our patient. Multiple patients with *RARS2*-related mitochondrial disorder without lactic acidosis have been reported, so this feature may be variable [3]. However, untargeted plasma metabolomics was analyzed in our patient. This study showed several abnormalities, including mild to moderately increased levels of lysophospholipids, phosphatidylcholines, and sphingomyelins (and their precursors–ceramide and dihydrosphingomyelins). Untargeted plasma metabolomics analyses have not been previously reported in *RARS2*-related mitochondrial disorder or in other aaRS disorders. Thus, it is difficult to determine the exact cause and prevalence of these abnormalities. One possible explanation, however, given the degree of generalized atrophy and volume loss on brain MRI, is that the elevations in serum of the lysophospholipid compounds may reflect a peripheral process that mirrors the loss of myelin in the cerebral white matter of the central nervous system (CNS). Although the precise etiology of these perturbations is not clearly understood, a previous publication on another mitochondrial syndrome, sideroblastic anemia with B-cell immunodeficiency, periodic fevers, and developmental delay (SIFD) (MIM #616084), caused by biallelic pathogenic variants in *TRNT1* also demonstrated abnormalities in sphingolipid and lysophospholipid metabolism in untargeted metabolomics analysis of plasma [24]. Furthermore, when the plasma metabolomics profiles of twenty-five patients with mitochondrial disorders were interrogated using high-throughput targeted semiquantitative analysis, elevations in the phospholipid ethanolamine were observed in patients with progressive external ophthalmoplegia (PEO) [25]. As lysophospholipid and sphingomyelin are precursors of phospholipid metabolites, our patient’s untargeted metabolomics analysis and those of prior publications suggest a common pathway that may be affected in several mitochondrial disorders. However, the reliability of these changes in lysophospholipid compounds needs to be validated in a larger cohort of patients with *RARS2*-related mitochondrial disorder and other mitochondrial disorders.

Dysmorphology in *RARS2*-related mitochondrial disorder has been observed in less than 20% of patients [3]. Multiple patients have been described with bitemporal narrowing, edematous hands and feet, full cheeks, and a high-arched or narrow palate [9, 26]. Such features are likely to be consequences of hypotonia, severe developmental delay, and FTT. Other common dysmorphic features include adducted thumbs, high nasal bridges, prominent ears, a low philtrum, and trichiasis [3]. Our patient had bilateral macrotia, and this finding has been noted in previous reports. However, he demonstrated other features that have not been previously reported including a depressed nasal bridge, downslanting palpebral fissures, retrognathia, and bilateral overriding second toes (Supplementary Table 1). Although dysmorphisms are not cardinal features and do not follow a specific pattern in this mitochondrial syndrome, it is worth noting that they could be part of the spectrum. Progressive microcephaly is also commonly observed in *RARS2*-related mitochondrial disorder. Our patient demonstrated a normal HC of 49.4 cm at four years and nine months (20th percentile, WHO Boys HC 0–5) (Supplementary Table 1). Therefore, this feature may not be present in all patients with this condition.

Collectively, our case demonstrates several unique features of *RARS2*-related mitochondrial disorder, including dysmorphic features, the absence of progressive microcephaly, the presence of a unique metabolomics signature, and the development of LGS, for which a KD may prove beneficial. Our report expands the known phenotypes of *RARS2*-related mitochondrial disorder and should help broaden the clinical spectrum of the disease. The use of a KD should be considered to help manage epilepsy in patients with this disorder. Moreover, elevations in analytes of phospholipid metabolism are observed in plasma untargeted metabolomics analysis performed in this patient. Further metabolomics analyses in additional patients with mitochondrial disease are needed to determine whether this could be a relevant metabolomics signature in this patient population.

### Supplementary Information

**Supplementary Material 1. **

## Data Availability

All data and materials supporting our findings are contained within the manuscript and in the additional data files (Additional File 1 FINAL.docx and Fig. 1 FINAL.docx). Additional File 1 FINAL.docx consists of a data table entitled “Supplementary Table 1: Genotype and phenotype comparison of patients with *RARS2*-related Mitochondrial Disorder”. Additional File 1 FINAL is a data table describing the various clinical and neuroradiologic findings of all published patients with *RARS2*-related Mitochondrial Disorder. The document format is.docx. The variants reported in the patient’s whole genome sequencing have been deposited in ClinVar by Baylor Genetics: *RARS2* c.36 + 1G > T, Variation ID 2442235, Accession SCV003835680.1; *RARS2* c.419 T > G, (p.Phe140Cys), Variation ID 215055, Accession SCV000992715.2.

## Abbreviations

RNA: Ribonucleic acid
tRNA: Transfer RNA
*RARS2*: *Arginyl-tRNA synthetase 2*
PCH: Pontocerebellar hypoplasia
DEE: Developmental and epileptic encephalopathy
LGS: Lennox-Gastaut Syndrome
Phe: Phenylalanine
Cys: Cysteine
KD: Ketogenic diet
MRI: Magnetic resonance imaging
HC: Head circumference
ACTH: Adrenocorticotropic Hormone
FTT: Failure to thrive
CDC: Centers for Disease Control and Prevention
WHO: World Health Organization
ASM: Anti-seizure medication
EEG: Electroencephalogram
BG: Baylor Genetics
GDF15: Growth differentiation factor 15
ATP: Adenosine triphosphate
aaRS: Aminoacyl tRNA synthetase
CADD: Combined annotation dependent depletion
gnomAD: Genome Aggregation Database
MAD: Modified Atkins Diet
*POLG*: *DNA polymerase gamma, catalytic subunit*
SLC25A12: *Solute carrier family 25 member 12*
*MT-TL1*: *Mitochondrially encoded tRNA leucine 1*
*MTO1*: *Mitochondrial tRNA translation optimization 1*
DT: Dietary therapies
*KARS2*: *Lysyl-tRNA synthetase 2*
SIFD: Sideroblastic anemia with B-cell immunodeficiency, periodic fevers, and developmental delay
*TRNT1*: *tRNA nucleotidyl transferase 1*
PEO: Progressive external ophthalmoplegia
mtDNA: mitochondrial DNA
ICU: Intensive care unit
CNV: Copy number variant
SNV: Single nucleotide variant
WGS: Whole genome sequencing
