# Supplementary material for: Expanded clinical phenotype and untargeted metabolomics analysis in RARS2-related mitochondrial disorder: a case report
Source: BMC Neurol. 2024 Mar 4;24:87. doi: 10.1186/s12883-024-03571-w (PMC10910770; doi:10.1186/s12883-024-03571-w)
Supplement: Supplementary file 1 — Supplementary Material 1. [file 12883_2024_3571_MOESM1_ESM.docx]

**Supplementary Table 1: Genotype and phenotype comparison of patients with *RARS2*-related Mitochondrial Disorder**

| **Article** | **Single nucleotide variant found in *RARS2*** | **Protein variant found in RARS2** | **Sex** | **Seizure semiology** | **Seizure onset** | **Anti-seizure medications** | **EEG** | **Neuroradiologic Findings** | **Dysmorphic features and other findings** |
| --- | --- | --- | --- | --- | --- | --- | --- | --- | --- |
| This article | c.36+1G>T,  c.419T>G | p.Phe140Cys | M | Focal motor, infantile spasms, tonic, myoclonic, myoclonic-tonic, Lennox-Gastaut Syndrome | 1 m | PB, ACTH, Prednisolone, VGB; ZNS, CLB, CBD, RFN, +Keto diet (significant improvement) | 12 m: Hypsarrhythmia with multifocal spikes.  4.5 y: Generalized paroxysmal fast activity in sleep, diffuse spike and slow waves | 4.5 y: Progressive generalized brain atrophy, PCH absent | Bilateral macrotia, downslanting palpebral fissures, depressed nasal bridge, retrognathia, and bilateral overriding second toes |
| Edvardson et al. 2007 | c.110 + 5A>G (homozygous) |  | F | Intractable, pharmacoresistant seizures, not further specified | 2 m | None | Generalized epileptic activity, attenuation over right hemisphere | 3 d: Vermian hypoplasia & cortical atrophy; 5 m: White matter and corpus callosum atrophy, mce, PCH present | Apnea, no dysmorphic features |
| Edvardson et al. 2007 | c.110 + 5A>G (homozygous) |  | M | No seizures reported | N/A | N/A | No | PCH NR | Died in crib at 7 w, dysmorphic features NR |
| Edvardson et al. 2007 | c.110 + 5A>G (homozygous) |  | F | Generalized seizures, not further specified | 4 m | NR | 3 w: Normal EEG | 3 m: Cortical/ white matter atrophy (greater in pons and cerebellum), mce, PCH present | No dysmorphic features |
| Nevanlinna et al. 2020 | c.795delA,  c.961C>T | p.Glu265Aspfs*16, p.Leu321Phe | M | Focal-to-bilateral- tonic-clonic, focal motor, focal with impaired awareness, myoclonic, non-convulsive status epilepticus | 6 w | NR | 3 m: Normal.  4 m: Multifocal discharges.  5 m: Modified hypsarrhythmia.  12 y: Status epilepticus, multifocal electrical activity | 4.5 m: delayed myelination, cerebellum nl., cortical atrophy, loss of white matter; 7 y: Severe cerebral & cerebellar atrophy, pons nl., myelination normalized, increased T2 signal in thalamus, mce, PCH absent | Open mouth, full cheeks, tented upper lip, bitemporal narrowing, mild edema of both hands |
| Glamuzina et al. 2012 | c.471_473delCAA, c.1211T>A | p.Lys158delLys, p.Met404Lys | F | Motor status epilepticus, unknown, tonic clonic | 4 w | Multiple, NR | 4 w: Burst suppression; 6 m: Slow, bifrontal discharges | 5 d, 6 w & 7 m: cortical (white matter), cerebellar atrophy & cerebellar cysts, optic n. atrophy, mce, PCH present | No dysmorphic features |
| Cassandrini et al. 2013 | c.25A>G,  c.1586 +3A>T | p.Ile9Val | M | Focal motor | 11 d | Multiple, NR | Slow, multifocal discharges | 1 m: Vermian hipoplasia; 2 y: Cortical atrophy, mce, PCH present | NR |
| Cassandrini et al. 2013 | c.25A>G,  c.1586 +3A>T | p.Ile9Val | M | Intractable, not further specified | 3 m | Multiple, NR | Slow, multifocal discharges | 3 m: Cerebral atrophy  3 y 4 m: Progressive cerebral atrophy, mce, PCH present | NR |
| Cassandrini et al. 2013 | c.734G>A,  c.1406G>A | p.Arg245Gln,  p. Arg469His | F | Motor status epilepticus, focal motor (multifocal) | 20 d | Multiple, NR | Slow, multifocal discharges | 3 y: Progressive cerebral atrophy and white matter loss, mce, PCH absent | NR |
| Cassandrini et al. 2013 | c.734G>A,  c.1406G>A | p.Arg245Gln,  p.Arg469His | F | Focal motor (clonic, multifocal) | 10 d | Multiple, NR | Slow, multifocal discharges | 2 y: Cortical atrophy, mce, PCH present | NR |
| Cassandrini et al. 2013 | c.35A>G,  c.721T>A | p.Gln12Arg, p.Trp241Arg | M | Intractable multifocal seizures | 20 d | Multiple, NR | Slow, multifocal discharges | 12 m & 2 y: Severe cortical atrophy, mce, PCH present | NR |
| Kastrissianakis et al. 2013 | c.773G>A,  c.1651-2A>G | p.Arg258His | F | Tonic clonic | 2 w | VPA, CLB, LEV, TPM | 14 w: Focal slowing (right hemisphere), multifocal discharges  16 m: Continuous spike and wave in sleep | 14 w: Subdural fluid collections, global cerebral atrophy.  22 m: Progressive cerebral & cerebellar atrophy, mce, PCH absent | No dysmorphic features |
| Kastrissianakis et al. 2013 | c.773G>A,  c.1651-2A>G | p.Arg258His | M | Focal motor (clonic, multifocal) | 1 d | PB, VGB, TPM, CLB | 1 d: Normal.  3 w: Burst suppression in sleep, multifocal discharges | 10 w CT scan: Cerebral atrophy  9 m: Progressive cerebral atrophy, PCH absent | No dysmorphic features |
| Rankin et al. 2010 | c.35A>G,  c.1024A>G | p.Gln12Arg, p.Met342Val | F | Myoclonic | 2 d | NR | 1 w: Discharges.  2 y: Generalized spike and waves | 14 m: Generalized cerebral atrophy, mce, PCH absent | Bitemporal narrowing, deep-set eyes, open mouth, edematous hands, full cheeks, edematous hands with tapering fingers |
| Ngoh et al. 2016 | c.472_474del, c.848T>A | p.Lys158del,  p.Leu283Gln | M | Clonic, epileptic spasms, tonic, generalized tonic clonic with status epilepticus | 5 w | PB, VBG, VPA, TPM, LEV, phenytoin, CLZ, +Keto diet (minor improvement) | 6 w: Normal.  8 m: Modified hypsarrhythmia.  11 m: Multifocal discharges | 7 m: Progressive mce, cerebral atrophy (white matter) and cerebellar atrophy, progressive mce, PCH absent | NR |
| Ngoh et al. 2016 | c.472_474del, c.848T>A | p.Lys158del,  p.Leu283Gln | M | Motor seizures (clonic), epileptic spasms, myoclonic | 8 w | PB, VGB, pyridoxal phosphate, multiple NR, +Keto diet (limited effectiveness) | 5 m: Modified hypsarrhythmia.  2.5 y: Multifocal discharges | 6 m: Progressive mce, cerebral atrophy (white matter) and cerebellar atrophy, progressive mce, PCH absent | NR |
| Nishri et al. 2016 | c.110+5A > G, c.878+5G > T |  | F | Focal motor (clonic, multifocal), myoclonic | 9 w | Multiple, NR,  +Keto diet (limited effectiveness) | 9 w: Normal; 4 m: Slowing, polyspike and slow waves, electrodecrement; 3 y: Multifocal discharges (migratory) | 2 m: Nl.; 16 m: Cerebral and cerebellar atrophy, mce, PCH absent | NR |
| Nishri et al. 2016 | c.110+5A > G, c.878+5G > T |  | M | Focal motor (clonic), myoclonic, tonic | 12 w | PB | 9 m: Multifocal discharges, polyspike and slow waves | 13 m: Cerebral atrophy (Diffuse gray matter atrophy), PCH absent | NR |
| Van Dijk et al. 2016  . | c.297+2T>G, c.1544A>G | p.Asp515Gly | F | Focal motor (clonic), motor status epilepticus | <12 w | Multiple, NR | 3 m: Multifocal discharges | 2 m: Cortical atrophy (white matter) and vermian hypoplasia; 6 m: Progressive cortical and cerebellar atrophy, subdural hygromas, progressive mce, MRS lactate, PCH absent | NR |
| Van Dijk et al. 2016 | c.452_454insC, c.1544A>G | p.Asn152Lysfs*40, p.Asp515Gly | M | Focal motor (clonic), motor status epilepticus | 3 m | Multiple, NR | 6 m: Multifocal epileptiform activity, frequent focal clonic seizures | 3 m: Cortical atrophy, T2 signal elevation in central and Parietooccipital cortex and white matter, PCH absent; MRS lactate | NR |
| Xu et al. 2020 | c.282_285del, c.773G>A | p.Arg94fs,  p. Arg258His | F | Myoclonic, convulsive status epilepticus, epileptic spasms | 19 d | PB, LEV, oxcarbazepine | 24 d: Burst suppression in wakefulness and sleep, myoclonic seizures in bursts | 27 d: Cerebral atrophy, abnormal white matter, small blood vessels bitemporally, PCH absent | NR |
| Minardi et al. 2020 | c.1026G>A c.1305+1G>A | p.Met342Ile | M | Epileptic Spasms, tonic, eyelid myoclonia | 8 m | VPA, LTG, CLZ | Bilateral focal epileptiform activities | No brain abnormalities reported | NR |
| Namavar et al. 2011 | c.35A>G,  c.110+5A>G | p.Gln12Arg | NR | NR | NR | NR | NR | PCH present | Died at 6 d, dysmorphic features NR |
| Joseph et al. 2014 | c.997C>G,  c.1432G>A | p.Arg333Gly, p.Gly478Arg | M | Seizures, not further specified | <1 m | NR | NR | Cerebral (white matter) and cerebellar atrophy, mce, subdural hygromas, PCH present | High arched palate and micrognathia, adducted thumbs |
| Joseph et al. 2014 | c.997C>G,  c.1432G>A | p.Arg333Gly, p.Gly478Arg | F | Seizures, not further specified | < 2 m | NR | NR | Fetal MRI 25 w: Cerebellar hypoplasia, cerebral (white matter) and cerebellar atrophy, mce, subdural hygromas, PCH present | Sloped forehead, micrognathia, adducted thumbs, knee contractures |
| Joseph et al. 2014 | c.997C>G,  c.1432G>A | p.Arg333Gly, p.Gly478Arg | F | Seizures, not further specified | 6 h | NR | NR | Fetal MRI 25 w: Cerebellar hypoplasia, cerebral (white matter) and cerebellar atrophy, mce, subdural hygromas, PCH present | Micrognathia, adducted thumbs |
| Lax et al. 2015 | c.1A>G,  c.613-3927C>T | p.Met1Val | F | No | N/A | N/A | N/A | None, PCH present | Lower back dermal pit, died as a neonate |
| Lax et al. 2015 | c.1A>G,  c.613-3927C>T | p.Met1Val | F | Yes | N/A | N/A | 2 d: Burst suppression | Fetal MRI 29 w: Ventricular hypertrophy; Fetal US: Cerebellar hypoplasia; MRI 8 d: Punctate foci in cerebral lobar white matter, PCH present | Small midface, wide lower face, depressed nasal bridge, generalized subcutaneous edema  Died at 14 d |
| Li et al. 2015 | c.-2A>G (homozygous) |  | M | Refractory focal epilepsy | 13 m | NR | NR | 9 m: Prominent subarachnoid spaces, PCH present | Clinodactyly adducted thumbs |
| Li et al. 2015 | c.-2A>G (homozygous) |  | F | No | N/A | N/A | N/A | Fetal MRI: No abnormalities; 7 m: PCH present | Proximal thumbs, large first toe |
| Alkhateeb et al. 2016 | c.1588C>T (homozygous) | p.His530Tyr | F | Seizures, not further specified | NR | NR | NR | NR | Downslanting PF |
| Alkhateeb et al. 2016 | c.1588C>T (homozygous) | p.His530Tyr | F | Seizures, not further specified | NR | NR | NR | NR | Downslanting PF |
| Alkhateeb et al. 2016 | c.1588C>T (homozygous) | p.His530Tyr | F | Seizures, not further specified | NR | NR | NR | NR | Downslanting PF |
| Shakya et al. 2019 | c.848T>A (homozygous) | p.Leu283Gln | F | Seizures, not further specified | NR | NR | NR | MRI: Normal, PCH absent | NR |
| Shakya et al. 2019 | c.848T>A (homozygous) | p.Leu283Gln | M | NR | NR | NR | NR | None, PCH absent | NR |
| Al Balushi et al. 2019 | c.633_636delAGAA,c.1113-21A>C | p.Glu212Glnfs*7, | F | Seizures, Status epilepticus | 1 d | NR | NR | 5 d: Vermian & superior cerebellar hypoplasia; 6 w: Dilation of anterior horns of LV, progressive supratentorial volume loss, mce, PCH absent | Died at 2 m, dysmorphic features NR |
| Mathew et al. 2018 | c.848T>A (homozygous) | p.Leu283Gln | M | Myoclonic seizures | Childhood | VPA, TPM | Diffuse slowing | 20 y: Normal, PCH absent | No dysmorphic features |
| Mathew et al. 2018 | c.848T>A (homozygous) | p.Leu283Gln | F | Myoclonic seizures | Childhood | VPA, lamotrigine, ZNS | Diffuse slowing | 24 y: Normal, PCH absent | No dysmorphic features |
| Gieldon et al. 2018 | c.16C>T,  c.1544A>G | p.Arg6Cys, p.Asp515Gly | F | Refractory epilepsy (not further specified), infantile spasms | 3 d | NR | NR | 1 w: Diffuse cerebral atrophy. 14 m: Progressive cerebral and supratentorial white matter atrophy, mild vermian atrophy, MRS with lactate and lipid elevations in basal ganglia, mce, PCH absent | NR |
| Pronicka et al. 2016 | c.622C>T, c.1026G>A | p.Gln208Ter, p.Met342Ile | M | NR | NR | NR | NR | NR | NR |
| Pronicka et al. 2016 | c.622C>T, c.1026G>A | p.Gln208Ter, p.Met342Ile | F | Infantile spasms | NR | NR | NR | Cerebellar atrophy, PCH NR | NR |
| Luhl et al. 2016 | c.392T > G (homozygous) | p.Phe131Cys | M | Symptomatic epilepsy, not further specified | 3 m | VGB, pyridoxine, folinic Acid, VPA, CLB, Sultiam, MP biotin, steroids | Continuous spikes and waves in sleep | 40 m: Thalamic and white matter atrophy, mce, PCH absent | NR |
| Luhl et al. 2016 | c.392T > G (homozygous) | p.Phe131Cys | F | NR | N/A | N/A | 5 d: Normal | 10 d: Normal, PCH absent | No dysmorphic features |
| Legati et al. 2016 | c.1A>G, c.1327T>C | p.Met1Val, p.Ser443Pro | M | Epilepsy, not further specified | NR | NR | NR | PCH present | NR |
| Sevinc et al. 2022 | c.1564G>A (homozygous) | p.Val522Ile | F | Epilepsy, not further specified | 3 m | LEV, PB | Epileptic encephalopathy with burst suppression | 8 d: Cerebral atrophy, gyrus expansion in subcortical white matter; 7 m: Marked loss and T2 hyperintensity of white matter, mce, PCH absent | Scooped and low ears, a low philtrum, prominent nasal bridge |
| Sevinc et al. 2022 | c.1564G>A (homozygous) | p.Val522Ile | F | Seizures, not further specified | 3 m | NR | NR | Cranial CT: Cerebral atrophic parenchyma, pontocerebral wide margins, mce, PCH absent | Micrognathia, closed fontanels, overriding metopic suture, prominent occipital bone, narrow forehead, low anterior hairline, thin upper lip |
| Valles-Ibanez et al. 2022 | c.1A>T,  c.848T>A | p.Met1Leu, p.Leu283Gln | F | Myoclonic, atonic, focal-to-bilateral- tonic-clonic, tonic, atypical absence, non-convulsive status epilepticus, motor status epilepticus | 2 y 2 m | Oral prednisolone, diazepam | 2 y 4 m: Multifocal discharges.  2 y 5 m & 2 y 9 m: Slow, multifocal discharges.  3 y 4 m & 4 y 5 m: Slow, multifocal discharges, generalized spike and wave, poly spike and slow wave, diffuse slowing | Thick rostrum/genu of corpus callosum, periventricular white matter hyperintensity, PCH present | NR |
| Valles-Ibanez et al. 2022 | c.36+5G>A, c.472_474delAAA | p.Lys158del | F | Atonic, Atypical Absence, Myoclonic- Atonic, Non-convulsive status epilepticus | 3 y | Steroids, 10 ASMs (NR), +Keto diet (limited effectiveness) | 2 y: Normal; 3 y: Generalized spike and waves; 8 y: Atypical absence seizures, generalized spike and wave, multifocal discharges, photo paroxysmal response | Generalized sulcal prominence, PCH absent | 30 Café-au-lait spots and axillary freckling |
| Lipponen et al. 2021 | c.773G>A | p.Arg258His, p.Ala369fs | F | Epilepsy, not further specified | NR | NR | NR | NR | NR |
| Zhang et al. 2022 | c.1060T>A,  c.1369G>A | p.Phe354Ile,  p.Gly457Arg | M | Severe epilepsy, seizure clusters | 6 m | VPA, MP, TPM | NR | NR | NR |
| Zhang et al. 2022 | c.1060T>A,  c.1369G>A | p.Phe354Ile,  p.Gly457Arg | F | Tonic, infantile spasms | 3 m | VPA, TPM | Atypical hypsarrhythmia, high-amplitude spike-and-wave complexes, low amplitude fast waves | Delayed myelination, PCH absent | Epicanthus, faint eyebrows, trichiasis, high nasal bridge, bulbous and high nose tip, cupid lips, prominent ears, bilateral frontal depression, and full cheeks |
| Zhang et al. 2022 | c.3G>C,  c.1060T>A | p. Met1Ile , p.Phe354Ile | F | Infantile spasms | 5 m | NR | Abnormal, suggestive of epilepsy | 5 m : Hydrocephalus, extracerebral space enlargement, PCH absent | No dysmorphic features |
| Zhang et al. 2022 | c.685C>T,  c.1210A>G | p.Arg229Ter*,*  p.Met404Val | M | Myoclonic, clonic | 3.5 m | Midazolam, PB, LEV, TPM | 3.5 m: Slow waves, spikes and spike-and-waves in left parietal, anterior temporal, bilateral occipital, middle and posterior temporal regions 4 m: Frequent single or continuous local myoclonic seizures and local clonic seizures in left central and parietal lobes, | None, PCH absent | Epicanthus inversus, faint eyebrows, high nasal bridge, bulbous and high nose tip, cupid lips, prominent ears, bilateral frontal depression, retracted lower jaw, and full cheeks |
| Zhang et al. 2022 | c.685C>T,  c.1210A>G | p.Arg229Ter ,  p.Met404Val | F | Seizures, not further specified | NR | NR | NR | NR | NR |
| Wu et al. 2020 | c.1210A>G,  c.622C>T | p.Met404Val, p.Gln208Ter | M | Tonic, focal motor | 3 m | NR | Continuous and periodic discharges of bilateral frontal sharp slow waves, no relationship with myoelectric burst | Enlarged ventricles, PCH absent | High arched palate, indirect inguinal hernia |
| Jiang et al. 2020 | c.1679G>A | p.Arg6His, p.Arg560His, | M | Seizures, early onset epileptic encephalopathy | 2 m | NR | NR | 4 m: Cerebral atrophy, mce, PCH absent | NR |
| Jiang et al. 2020 |  | p.Phe5Ser, p.Arg254Trp | F | Seizures, early onset epileptic encephalopathy | 29 d | NR | NR | 4 m: Cerebral atrophy, mce, PCH absent | NR |
| Bendeck et al. 2022 | c.16C>T,  c.1679G>A | p.Arg6Cys, p.Arg560His | F | Tonic clonic, convulsive status epilepticus | 4 m | LEV, PB | 4 m: Normal.  1 y: Multifocal discharges | 1 yr: Cortical atrophy, thinned corpus callosum, delayed myelination in occipital/frontal lobes, vermian hypoplasia, PCH absent | NR |
| Nuovo et al. 2022 | c.1A>G,  c.1327T>C | p.Met1Val, p.Ser443Pro | M | Myoclonic seizures | NR | NR | NR | mce, other features NR | Cryptorchidism |
| Obeid et al. 2018 | NR | NR | F | Non-convulsive status epilepticus, focal motor, behavioral arrest, focal motor status epilepticus | 2 m | PB, oxcarbazepine, lacosamide, TPM, ZNS, LEV, fosphenytoin, midazolam | Focal seizures, multifocal sharp waves, disorganized background | 2 m: Excessive cytotoxic edema and cerebral atrophy, mce, PCH present | NR |
| Zhang et al. 2018 | c.991A>G, c.1718C>T | p.Ile331Val, p.Thr573Ile | M | Multifocal myoclonic, focal | 3 m | VPA, LEV, TPM, PB, CLZ, +Keto diet (not tolerated) | Diffuse slow waves, multifocal spike and fast waves, focal seizures | 4 & 5 m: High DWI signal in frontal and parietal cortex  7 m: Cerebral cortex atrophy, subdural effusions, corpus callosum white matter depletion, mce, PCH absent | NR |
| Gong et al. 2021 | c.991A>G, c.1718C>T | p.Ile331Val, p.Thr573Ile | NR | Focal, atypical absence, electrical status epilepticus of sleep | 3 m | TPM, PB | Generalized spike and waves at onset; Repeat: spike and sharp waves in Rolandic region | Cerebral atrophy, mce, PCH absent | NR |
| Roux et al. 2021 | c.29C>T,  c.298-1G>A | p.Ala10Val, | NR | Epileptic encephalopathy, not further specified | NR | NR | NR | 1 m: Early white matter changes with mild diffuse global brain atrophy, PCH present | NR |
| Roux et al. 2021 | c.442A>G, c.472_474del | p.Thr148Ala,  p.Lys158del | NR | No | No | NR | NR | 10 m: Global brain atrophy, PCH present | NR |
| Roux et al. 2021 | c.35A>G,  c.601C>G | p.Gln12Arg, p.His201Asp | NR | Epileptic encephalopathy, not further specified | NR | NR | NR | 3 d: Early white matter changes with mild diffuse global brain atrophy, PCH present | NR |
| Roux et al. 2021 | c.965A>G,  c.1564G>A | p. Tyr322Cys, p.Val522Ile | NR | Epilepsy, not further specified | NR | NR | NR | None, PCH absent | NR |
| Fresard et al. 2018 | c.419T>G, c.1612delA | p.Phe140Cys, p.Thr538fs | F | Epilepsy, not further specified | Neonatal onset | NR | NR | Mce, other features NR | Progressive scoliosis |
| Fresard et al. 2018 | c.419T>G, c.1612delA | p.Phe140Cys, p.Thr538fs | F | Epilepsy, not further specified | Neonatal onset | NR | NR | Mce, other features NR | Progressive scoliosis |

**Legend:**

| NR- not reported | MP- Methylprednisone | LV- lateral ventricle | |
| --- | --- | --- | --- |
| UK- unknown | VGB- Vigabatrin | n- nerve | |
| N/A- not available | CLB- Clobazam |  | |
| m- months | VPA- Valproic acid |  | |
| w- weeks | LEV- Leviteracetam |  |  |
| d- day | PB- Phenobarbital |  |  |
| y-year | TPM- Topiramate |  | |
| fs- frameshift | ZNS- Zonisamide |  | |
| PCH- pontocerebellar hypoplasia | RFN- Rufinamide |  | |
| mce- microcephaly | CBD- Cannibdiol |  | |
| CSF- cerebrospinal fluid | CLZ- Clonazepam |  | |
| Nl.- normal | Keto diet- Ketogenic diet |  |  |

**Supplementary References:**

1. Edvardson S, Shaag A, Kolesnikova O, Gomori JM, Tarassov I, Einbinder T, Saada A, Elpeleg O. Deleterious mutation in the *mitochondrial arginyl-transfer RNA synthetase* gene is associated with pontocerebellar hypoplasia. Am J Hum Genet. 2007 Oct;81(4):857-62. doi: 10.1086/521227. Epub 2007 Aug 24. PMID: 17847012; PMCID: PMC2227936.
2. Nevanlinna V, Konovalova S, Ceulemans B, Muona M, Laari A, Hilander T, Gorski K, Valanne L, Anttonen AK, Tyynismaa H, Courage C, Lehesjoki AE. A patient with pontocerebellar hypoplasia type 6: Novel *RARS2* mutations, comparison to previously published patients and clinical distinction from PEHO syndrome. Eur J Med Genet. 2020 Mar;63(3):103766. doi: 10.1016/j.ejmg.2019.103766. Epub 2019 Sep 16. PMID: 31536827.
3. Glamuzina E, Brown R, Hogarth K, Saunders D, Russell-Eggitt I, Pitt M, de Sousa C, Rahman S, Brown G, Grunewald S. Further delineation of pontocerebellar hypoplasia type 6 due to mutations in the gene encoding *mitochondrial arginyl-tRNA synthetase*, *RARS2*. J Inherit Metab Dis. 2012 May;35(3):459-67. doi: 10.1007/s10545-011-9413-6. Epub 2011 Nov 16. PMID: 22086604.
4. Cassandrini D, Cilio MR, Bianchi M, Doimo M, Balestri M, Tessa A, Rizza T, Sartori G, Meschini MC, Nesti C, Tozzi G, Petruzzella V, Piemonte F, Bisceglia L, Bruno C, Dionisi-Vici C, D'Amico A, Fattori F, Carrozzo R, Salviati L, Santorelli FM, Bertini E. Pontocerebellar hypoplasia type 6 caused by mutations in *RARS2*: definition of the clinical spectrum and molecular findings in five patients. J Inherit Metab Dis. 2013 Jan;36(1):43-53. doi: 10.1007/s10545-012-9487-9. Epub 2012 May 8. PMID: 22569581.
5. Kastrissianakis K, Anand G, Quaghebeur G, Price S, Prabhakar P, Marinova J, Brown G, McShane T. Subdural effusions and lack of early pontocerebellar hypoplasia in siblings with *RARS2* mutations. Arch Dis Child. 2013 Dec;98(12):1004-7. doi: 10.1136/archdischild-2013-304308. Epub 2013 Sep 18. PMID: 24047924.
6. Rankin J, Brown R, Dobyns WB, Harington J, Patel J, Quinn M, Brown G. Pontocerebellar hypoplasia type 6: A British case with PEHO-like features. Am J Med Genet A. 2010 Aug;152A(8):2079-84. doi: 10.1002/ajmg.a.33531. PMID: 20635367.
7. Ngoh A, Bras J, Guerreiro R, Meyer E, McTague A, Dawson E, Mankad K, Gunny R, Clayton P, Mills PB, Thornton R, Lai M, Forsyth R, Kurian MA. *RARS2* mutations in a sibship with infantile spasms. Epilepsia. 2016 May;57(5):e97-e102. doi: 10.1111/epi.13358. Epub 2016 Apr 8. PMID: 27061686; PMCID: PMC4864753.
8. Nishri D, Goldberg-Stern H, Noyman I, Blumkin L, Kivity S, Saitsu H, Nakashima M, Matsumoto N, Leshinsky-Silver E, Lerman-Sagie T, Lev D. *RARS2* mutations cause early onset epileptic encephalopathy without ponto-cerebellar hypoplasia. Eur J Paediatr Neurol. 2016 May;20(3):412-7. doi: 10.1016/j.ejpn.2016.02.012. Epub 2016 Mar 2. PMID: 26970947.
9. van Dijk T, van Ruissen F, Jaeger B, Rodenburg RJ, Tamminga S, van Maarle M, Baas F, Wolf NI, Poll-The BT. *RARS2* Mutations: Is Pontocerebellar Hypoplasia Type 6 a Mitochondrial Encephalopathy? JIMD Rep. 2017;33:87-92. doi: 10.1007/8904_2016_584. Epub 2016 Sep 29. PMID: 27683254; PMCID: PMC5413457.
10. Xu Y, Wu BB, Wang HJ, Zhou SZ, Cheng GQ, Zhou YF. A term neonate with early myoclonic encephalopathy caused by *RARS2* gene variants: a case report. Transl Pediatr. 2020 Oct;9(5):707-712. doi: 10.21037/tp-20-110. PMID: 33209735; PMCID: PMC7658767.
11. Minardi R, Licchetta L, Baroni MC, Pippucci T, Stipa C, Mostacci B, Severi G, Toni F, Bergonzini L, Carelli V, Seri M, Tinuper P, Bisulli F. Whole-exome sequencing in adult patients with developmental and epileptic encephalopathy: It is never too late. Clin Genet. 2020 Nov;98(5):477-485. doi: 10.1111/cge.13823. Epub 2020 Sep 1. PMID: 32725632.
12. Namavar Y, Barth PG, Kasher PR, van Ruissen F, Brockmann K, Bernert G, Writzl K, Ventura K, Cheng EY, Ferriero DM, Basel-Vanagaite L, Eggens VR, Krägeloh-Mann I, De Meirleir L, King M, Graham JM Jr, von Moers A, Knoers N, Sztriha L, Korinthenberg R; PCH Consortium; Dobyns WB, Baas F, Poll-The BT. Clinical, neuroradiological and genetic findings in pontocerebellar hypoplasia. Brain. 2011 Jan;134(Pt 1):143-56. doi: 10.1093/brain/awq287. Epub 2010 Oct 15. PMID: 20952379; PMCID: PMC9136852.
13. Joseph JT, Innes AM, Smith AC, Vanstone MR, Schwartzentruber JA, Bulman DE, Majewski J, Daza RA, Hevner RF, Michaud J, Boycott KM; FORGE Canada Consortium. Neuropathologic features of pontocerebellar hypoplasia type 6. J Neuropathol Exp Neurol. 2014 Nov;73(11):1009-25. doi: 10.1097/NEN.0000000000000123. PMID: 25289895.
14. Lax NZ, Alston CL, Schon K, Park SM, Krishnakumar D, He L, Falkous G, Ogilvy-Stuart A, Lees C, King RH, Hargreaves IP, Brown GK, McFarland R, Dean AF, Taylor RW. Neuropathologic Characterization of Pontocerebellar Hypoplasia Type 6 Associated With Cardiomyopathy and Hydrops Fetalis and Severe Multisystem Respiratory Chain Deficiency due to Novel *RARS2* Mutations. J Neuropathol Exp Neurol. 2015 Jul;74(7):688-703. doi: 10.1097/NEN.0000000000000209. PMID: 26083569; PMCID: PMC4470523.
15. Li Z, Schonberg R, Guidugli L, Johnson AK, Arnovitz S, Yang S, Scafidi J, Summar ML, Vezina G, Das S, Chapman K, del Gaudio D. A novel mutation in the promoter of *RARS2* causes pontocerebellar hypoplasia in two siblings. J Hum Genet. 2015 Jul;60(7):363-9. doi: 10.1038/jhg.2015.31. Epub 2015 Mar 26. PMID: 25809939; PMCID: PMC5537600.
16. Alkhateeb AM, Aburahma SK, Habbab W, Thompson IR. Novel mutations in *WWOX*, *RARS2*, and *C10orf2* genes in consanguineous Arab families with intellectual disability. Metab Brain Dis. 2016 Aug;31(4):901-7. doi: 10.1007/s11011-016-9827-9. Epub 2016 Apr 28. PMID: 27121845.
17. Shakya S, Kumari R, Suroliya V, Tyagi N, Joshi A, Garg A, Singh I, Kalikavil Puthanveedu D, Cherian A, Mukerji M, Srivastava AK, Faruq M. Whole exome and targeted gene sequencing to detect pathogenic recessive variants in early onset cerebellar ataxia. Clin Genet. 2019 Dec;96(6):566-574. doi: 10.1111/cge.13625. Epub 2019 Sep 1. PMID: 31429931.
18. Al Balushi A, Matviychuk D, Jobling R, Salomons GS, Blaser S, Mercimek-Andrews S. Phenotypes and genotypes of mitochondrial aminoacyl-tRNA synthetase deficiencies from a single neurometabolic clinic. JIMD Rep. 2019 Dec 18;51(1):3-10. doi: 10.1002/jmd2.12079. PMID: 32071833; PMCID: PMC7012735.
19. Mathew T, Avati A, D'Souza D, Therambil M. Expanding spectrum of *RARS*2 gene disorders: Myoclonic epilepsy, mental retardation, spasticity, and extrapyramidal features. Epilepsia Open. 2018 Mar 23;3(2):270-275. doi: 10.1002/epi4.12108. PMID: 29881806; PMCID: PMC5983106.
20. Gieldon L, Mackenroth L, Kahlert AK, Lemke JR, Porrmann J, Schallner J, von der Hagen M, Markus S, Weidensee S, Novotna B, Soerensen C, Klink B, Wagner J, Tzschach A, Jahn A, Kuhlee F, Hackmann K, Schrock E, Di Donato N, Rump A. Diagnostic value of partial exome sequencing in developmental disorders. PLoS One. 2018 Aug 9;13(8):e0201041. doi: 10.1371/journal.pone.0201041. Erratum in: PLoS One. 2020 Sep 24;15(9):e0239959. Erratum in: PLoS One. 2022 Jun 22;17(6):e0270541. PMID: 30091983; PMCID: PMC6084857.
21. Pronicka E, Piekutowska-Abramczuk D, Ciara E, Trubicka J, Rokicki D, Karkucińska-Więckowska A, Pajdowska M, Jurkiewicz E, Halat P, Kosińska J, Pollak A, Rydzanicz M, Stawinski P, Pronicki M, Krajewska-Walasek M, Płoski R. New perspective in diagnostics of mitochondrial disorders: two years' experience with whole-exome sequencing at a national paediatric centre. J Transl Med. 2016 Jun 12;14(1):174. doi: 10.1186/s12967-016-0930-9. PMID: 27290639; PMCID: PMC4903158.
22. Lühl S, Bode H, Schlötzer W, Bartsakoulia M, Horvath R, Abicht A, Stenzel M, Kirschner J, Grünert SC. Novel homozygous *RARS2* mutation in two siblings without pontocerebellar hypoplasia - further expansion of the phenotypic spectrum. Orphanet J Rare Dis. 2016 Oct 21;11(1):140. doi: 10.1186/s13023-016-0525-9. PMID: 27769281; PMCID: PMC5073905.
23. Legati A, Reyes A, Nasca A, Invernizzi F, Lamantea E, Tiranti V, Garavaglia B, Lamperti C, Ardissone A, Moroni I, Robinson A, Ghezzi D, Zeviani M. New genes and pathomechanisms in mitochondrial disorders unraveled by NGS technologies. Biochim Biophys Acta. 2016 Aug;1857(8):1326-1335. doi: 10.1016/j.bbabio.2016.02.022. Epub 2016 Mar 8. PMID: 26968897.
24. Sevinç S, İnci A, Ezgü FS, Eminoğlu FT. A Patient with a Novel *RARS2* Variant Exhibiting Liver Involvement as a New Clinical Feature and Review of Literature. Mol Syndromol. 2022 May;13(3):226-234. doi: 10.1159/000519604. Epub 2022 Feb 1. PMID: 35707589; PMCID: PMC9149545.
25. de Valles-Ibáñez G, Hildebrand MS, Bahlo M, King C, Coleman M, Green TE, Goldsmith J, Davis S, Gill D, Mandelstam S, Scheffer IE, Sadleir LG. Infantile-onset myoclonic developmental and epileptic encephalopathy: A new *RARS2* phenotype. Epilepsia Open. 2022 Mar;7(1):170-180. doi: 10.1002/epi4.12553. Epub 2021 Nov 18. PMID: 34717047; PMCID: PMC8886097.
26. Lipponen J, Helisalmi S, Raivo J, Siitonen A, Doi H, Rusanen H, Lehtilahti M, Ryytty M, Laakso M, Tanaka F, Majamaa K, Kytövuori L. Molecular epidemiology of hereditary ataxia in Finland. BMC Neurol. 2021 Oct 2;21(1):382. doi: 10.1186/s12883-021-02409-z. PMID: 34600502; PMCID: PMC8487109.
27. Zhang Y, Yu Y, Zhao X, Xu Y, Chen L, Li N, Yao R, Wang J, Yu T. Novel *RARS2* Variants: Updating the Diagnosis and Pathogenesis of Pontocerebellar Hypoplasia Type 6. Pediatr Neurol. 2022 Jun;131:30-41. doi: 10.1016/j.pediatrneurol.2022.04.002. Epub 2022 Apr 15. PMID: 35468344.
28. Wu TH, Peng J, Zhang CL, Wu LW, Yang LF, Peng P, Pang N, Yin F, He F. [Mutations in aminoacyl-tRNA synthetase genes: an analysis of 10 cases]. Zhongguo Dang Dai Er Ke Za Zhi. 2020 Jun;22(6):595-601. Chinese. doi: 10.7499/j.issn.1008-8830.1912040. PMID: 32571458; PMCID: PMC7390216.
29. Jiang HF, Deng J, Fang F, Li H, Wang XH, Dai LF. [Early onset epileptic encephalopathy caused by mitochondrial arginyl-tRNA synthetase gene deficiency: report of two cases and literature review]. Zhonghua Er Ke Za Zhi. 2020 Nov 2;58(11):893-899. Chinese. doi: 10.3760/cma.j.cn112140-20200716-00729. PMID: 33120460.
30. Bendeck JL, Villamizar I, Prieto C, Celis LG. Mutación heterocigota, autosómica recesiva del gen *RARS2* en una paciente colombiana de padres no consanguíneos [Autosomal recessive heterocygote mutation of the *RARS2* gene in a Colombian patient with non- consanguineous parents]. Arch Argent Pediatr. 2022 Feb;120(1):e39-e48. Spanish. doi: 10.5546/aap.2022.e39. Epub 2022 Jan 1. PMID: 35068129.
31. Nuovo S, Micalizzi A, Romaniello R, Arrigoni F, Ginevrino M, Casella A, Serpieri V, D'Arrigo S, Briguglio M, Salerno GG, Rossato S, Sartori S, Leuzzi V, Battini R, Ben-Zeev B, Graziano C, Mirabelli Badenier M, Brankovic V, Nardocci N, Spiegel R, Petković Ramadža D, Vento G, Marti I, Simonati A, Dipresa S, Freri E, Mazza T, Bassi MT, Bosco L, Travaglini L, Zanni G, Bertini ES, Vanacore N, Borgatti R, Valente EM. Refining the mutational spectrum and gene-phenotype correlates in pontocerebellar hypoplasia: results of a multicentric study. J Med Genet. 2022 Apr;59(4):399-409. doi: 10.1136/jmedgenet-2020-107497. Epub 2021 Mar 5. PMID: 34085948.
32. Obeid R, Sogawa Y, Naik M, Goldstein A, Gropman A, Asato M. A Newborn With Hyperlactatemia and Epileptic Encephalopathy. Semin Pediatr Neurol. 2018 Jul;26:104-107. doi: 10.1016/j.spen.2017.05.004. Epub 2017 May 23. PMID: 29961496.
33. Zhang J, Zhang Z, Zhang Y, Wu Y. Distinct magnetic resonance imaging features in a patient with novel *RARS2* mutations: A case report and review of the literature. Exp Ther Med. 2018 Jan;15(1):1099-1104. doi: 10.3892/etm.2017.5491. Epub 2017 Nov 10. PMID: 29434700; PMCID: PMC5772945.
34. Gong P, Xue J, Jiao X, Zhang Y, Yang Z. Genetic Etiologies in Developmental and/or Epileptic Encephalopathy With Electrical Status Epilepticus During Sleep: Cohort Study. Front Genet. 2021 Apr 8;12:607965. doi: 10.3389/fgene.2021.607965. PMID: 33897753; PMCID: PMC8060571.
35. Roux CJ, Barcia G, Schiff M, Sissler M, Levy R, Dangouloff-Ros V, Desguerre I, Edvardson S, Elpeleg O, Rötig A, Munnich A, Boddaert N. Phenotypic diversity of brain MRI patterns in mitochondrial aminoacyl-tRNA synthetase mutations. Mol Genet Metab. 2021 Jun;133(2):222-229. doi: 10.1016/j.ymgme.2021.04.004. Epub 2021 Apr 21. PMID: 33972171.
36. Frésard L, Smail C, Ferraro NM, Teran NA, Li X, Smith KS, Bonner D, Kernohan KD, Marwaha S, Zappala Z, Balliu B, Davis JR, Liu B, Prybol CJ, Kohler JN, Zastrow DB, Reuter CM, Fisk DG, Grove ME, Davidson JM, Hartley T, Joshi R, Strober BJ, Utiramerur S; Undiagnosed Diseases Network; Care4Rare Canada Consortium; Lind L, Ingelsson E, Battle A, Bejerano G, Bernstein JA, Ashley EA, Boycott KM, Merker JD, Wheeler MT, Montgomery SB. Identification of rare-disease genes using blood transcriptome sequencing and large control cohorts. Nat Med. 2019 Jun;25(6):911-919. doi: 10.1038/s41591-019-0457-8. Epub 2019 Jun 3. PMID: 31160820; PMCID: PMC6634302.
